# Case of Aneurism of the Innominate Artery, Presented by Dr. L. A. Stimpson, at the New York Surgical Society

**Published:** 1880-09

**Authors:** 


CASE OF ANEURISM OF THE INNOMINATE ARTERY. PRESENTED BY DR. L. A. STIMSON, AT THE NEW YORK SURGICAL SOCIETY.

An Irishman, 34 years of age, presented himself at Bellevue Hospital about the 1st of January, 1880, for treatment for pain in the right shoulder, that had begun in the preceding March, had gradually increased in severity, and finally became so severe as to cause him to stop his work. Diagnosis of innominate aneurism was made. Treatment by simultaneous ligation of the common carotid and subclavian arteries was suggested, and the operation was performed on the 22d of January. The wound healed promptly and kindly, and no accident occurred in the course of the recovery. For two or three weeks the tumor remained unchanged externally; then it began to diminish in size, the murmer disappeared, and it solidified. Previous to the operation the lateral diameter of the tumor was three inches, and its height above the clavicle one and one-half inches. Its reduced diameter was about one inch, and its height above the clavicle half an inch. A distinct pulsation remained. Whether it was from within or was communicated, Dr. Stimson did not feel certain, and asked the opinion of the Society upon that point. There had been no recurrence of pain in the shoulder except once, two weeks after the operation, and it disappeared promptly after the application of a blister. In the progress of the case there was an extremely rapid pulse, reaching 115 to 120, without fever or pain to explain its occurrence. So far as he knew, it was the seventeenth complete case.—*Medical Record.*

